# Neuroendocrine and immune pathways from pre- and perinatal stress to substance abuse

**DOI:** 10.1016/j.ynstr.2018.09.004

**Published:** 2018-09-17

**Authors:** Sarah R. Horn, Leslie E. Roos, Elliot T. Berkman, Philip A. Fisher

**Affiliations:** University of Oregon, Department of Psychology, 1227 University of Oregon, Eugene, OR, 97402, USA

**Keywords:** Prenatal stress, Perinatal stress, Substance abuse, Neuroendocrine, Immune, Translational neuroscience

## Abstract

Early life adversity is a documented risk factor for substance abuse and addiction. The pre- and perinatal period (i.e., from implantation, through pregnancy, to 6 months of age) is a critical period marked by high biological plasticity and vulnerability, making perinatal stress a particularly robust form of adversity. The neuroendocrine and immune systems are key mechanisms implicated in the transmission of addiction risk. We review animal and human studies that provide preliminary evidence for links between perinatal stress, neuroendocrine and immune dysregulation, and risk for substance abuse and addiction. *A translational neuroscience* perspective is employed to elucidate pre- and perinatally-induced biological mechanisms linked to addiction and discuss implications for prevention and intervention efforts. Significant evidence supports associations between pre- and perinatal stress and dysregulation of the hypothalamic-pituitary-adrenal axis and immune systems as well as links between neuroendocrine/immune functioning and addiction risk. More work is needed to explicitly examine the interplay between pre- and perinatal stress and neuroendocrine/immune disruptions that together heighten substance abuse risk. Future work is needed to fully understand how pre- and perinatal stress induces biological alterations to predispose individuals to higher risk for addiction. Such knowledge will strengthen theoretically-driven and empirically-supported prevention efforts for substance abuse and addiction.

## 1 Neuroendocrine and immune pathways from pre- and perinatal stress to substance abuse

Approximately 21 million United States adults suffer from a substance use disorder (SUD), making substance abuse and addiction among the most urgent and costly public health problems (US Department of Health and Human Services, Office of the Surgeon General, 2016). Delineating the underlying factors that contribute to SUDs and addiction is key to mitigating the impact of addiction on individuals and communities. Early life stress is a well-documented risk factor for drug and alcohol abuse and addiction (Enoch, 2011; Stone et al., 2012). As such, elucidating the underlying neurobiological mechanisms linking early life stress and substance abuse has potential to inform and optimize prevention and treatment strategies.

**Fig. 1:**
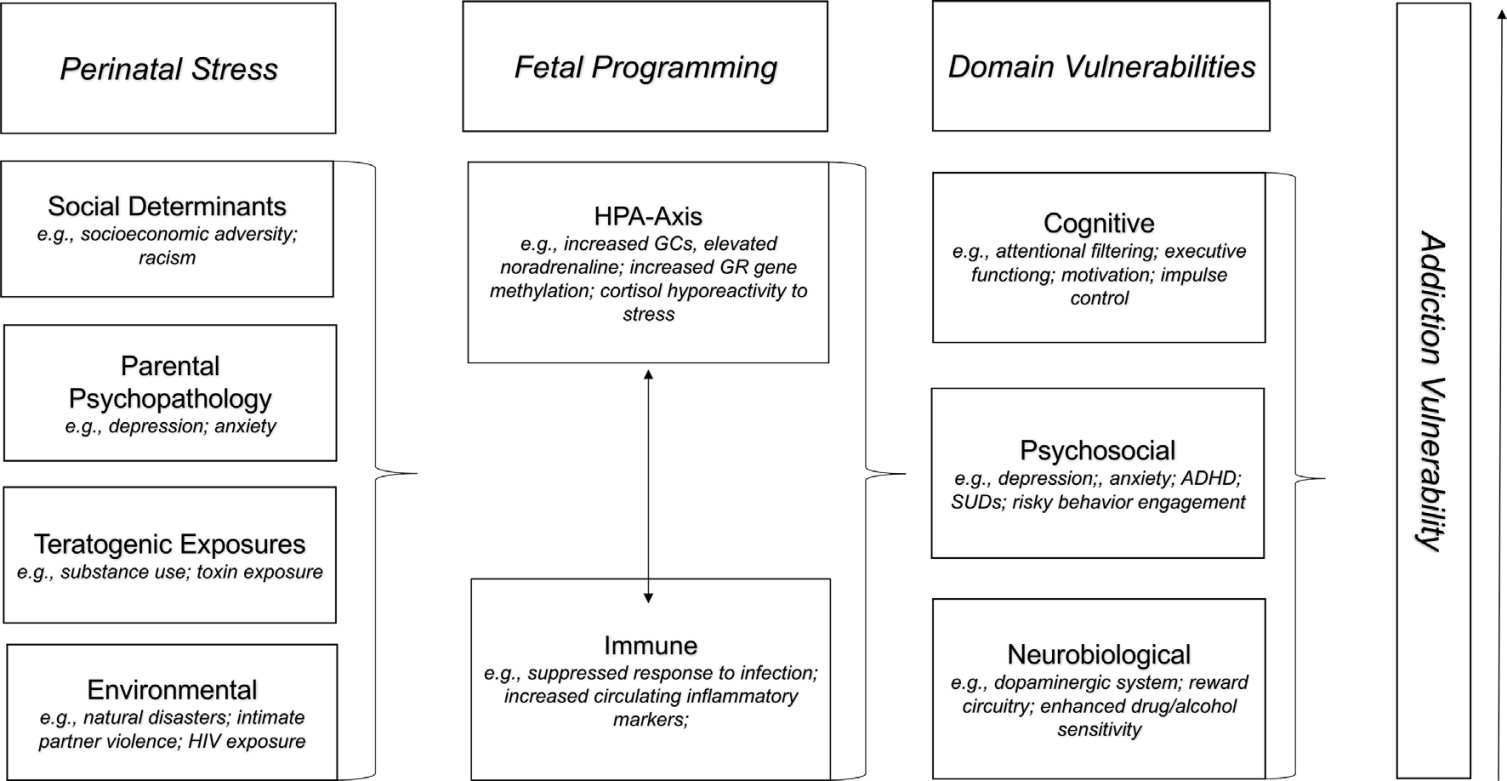
Conceptual model of neuroendocrine and immune pathways from perinatal stress to addiction vulnerability.

The effects of stress on later health-risking behavior can begin as early as the pre- and perinatal period (i.e., from implantation, through pregnancy, to 6 months of age), a critical period of rapid fetal brain development marked by high biological plasticity and vulnerability (Lupien et al., 2009; Provençal and Binder, 2015). A complex cycle of intergenerational transmission exists, in which mothers with substance-use problems, having themselves experienced higher rates of childhood abuse, neglect, and prenatal substance exposure, are more likely to expose their offspring to similar stressors during the pre/perinatal period than mothers without substance-use problems. This cycle contributes to risk for developing substance addiction issues later in life (Cash and Wilke, 2003). Through translational neuroscience, animal and human studies have begun to establish how early forms of adversity become “biologically embedded,” providing insight into mechanistic pathways that link pre/perinatal stress to future maladaptive outcomes, such as substance abuse and addiction (Miller et al., 2011). Notably, the neuroendocrine and immune systems are believed to be key mechanisms in the transmission of addiction and psychopathology risk related to acute and chronic stressors during the pre- and perinatal period (Enoch, 2011; Koob and Le Moal, 2001; Mayes and Suchman, 2006). Extant etiological models for substance abuse and addiction have limited focus thus far on the earliest developmental stages (Eiden et al., 2016), in which endocrine and immune processes may serve as potential mechanisms for future addiction-related risk. A proposed conceptual model details how pre- and perinatal stress may confer risk for addiction vulnerability through neuroendocrine and immune pathways (see Fig. 1).

This review synthesizes animal and human literature to implicate how exposure to stress during gestation and infancy impacts neuroendocrine and immune pathways to confer risk for substance abuse and addiction. First, we review animal and human studies on three broad categories of prenatal stress: maternal stress (e.g., psychosocial stress, psychopathology), stress in the context of laboratory studies of glucocorticoid administration, and stress arising from prenatal substance exposure. In these sections, we integrate evidence on the role of the hypothalamic-pituitary-adrenal (HPA) axis. We then transition to review links between pre- and perinatal stress, offspring substance use, and immune system function. This includes a review of relevant animal studies on immune activation, viral infection, and prenatal drug administration. We then discuss links between stress, prenatal drug use, and inflammation in human studies. We include a brief section on the role of perinatal human immunodeficiency virus (HIV) exposure as a naturalistic window to understanding immune-related stressors on vulnerability to substance misuse in offspring. For each section, we first review links between pre- and perinatal stressors and substance abuse vulnerability. We then review literature on the role of the HPA-axis or immune system in these pathways. Due to the limited extant literature on these topics, stressor types have been grouped together. Further, a comprehensive review of the entire literature on each substance was outside the scope of the present paper. Table 1 includes definitions of the most common type of animal stress paradigms with their corresponding human analog.

**Table 1:** Examples of animal models of pre- and perinatal stress and human analogs.

Name	Description	Human Analog
Prenatal substance exposure	Injection of substance (e.g., cocaine, morphine) into mother	Prenatal substance exposure
Synthetic GC exposure	Injection of synthetic glucocorticoid into mother	Glucocorticoid use during pregnancy
Food restriction paradigm	Restrict mother access to food	Malnutrition
Restraint and immobilization stress paradigm	Mother kept in cylindrical tube or restrained with adhesive tape	Prenatal anxiety
Immune activation	synthetic double strand RNA polyriboinosinic-polyribocytidilic acid (poly I:C) administered into mother	Prenatal viral infection
Maternal separation	Offspring exposed to maternal absence during first postnatal weeks	Postnatal neglect

Throughout the review, we employ a *translational neuroscience* perspective on the problem of intergenerational transmission of substance use and addiction. This perspective encourages a nuanced understanding of how these pathways link stress during early developmental stages to substance abuse across the lifespan (Fisher and Berkman, 2015). Specifically, translational neuroscience aims to pinpoint causal and moderating biological factors with the goal of leveraging this knowledge to the optimization of prevention and intervention strategies (Fisher and Berkman, 2015). The objective of translational neuroscience research is to not only to elucidate the multitudinous impacts of early life stress on these individual mechanisms that confers risk for substance abuse, but also to examine the interplay amongst them. We close by discussing implications for prevention and intervention strategies for addiction.

### 1.1 Early experiences, the HPA axis, and pathways to substance abuse

Due the semi-permeable nature of the placenta, high levels of maternal stress and associated physiological alterations in neuroendocrine and immune systems produce changes in the fetal environment (Barrett et al., 2017). This process is referred to as fetal programming (Glover et al., 2010; Kapoor et al., 2008). While fetal programming can be detrimental in many modern contexts, it may have served a useful purpose earlier in human evolutionary history, such as resource conservation in harsh environments (Seckl and Holmes, 2007). Stress effects via fetal programming are considered a conduit for children's later vulnerability to a range of mental and physical health detriments, including substance abuse and addiction. One of the primary factors underlying the pathway from fetal development to adult-onset disorders are glucocorticoids (GCs), cortisol in humans, which are key mechanisms of HPA axis programming (Davis and Sandman, 2010). Glucocorticoids are a particularly potent chemical signal due to their far-reaching impacts across every major regulatory system (e.g., immune, gut, autonomic nervous system, neural) (McEwen, 2013). Animal models have illustrated that the neuroendocrine and immune effects of profound stress are principally mediated through GC pathways (Lupien et al., 2009). However, evidence from human studies is more limited.

The neuroendocrine system is central to establishing and maintaining homeostatic processes as well as the short-term mobilization of biological resources in response to acute stress. The system is regulated by the release of hypothalamic corticotrophin-releasing hormone (CRH), which stimulates adrenocorticotropic hormone (ACTH) from the pituitary gland and, in turn, results in the production and release of cortisol from the adrenal cortex. By two-months of age, a diurnal pattern of cortisol release begins to develop, with low levels of cortisol over-night, which decline throughout the day (de Weerth et al., 2003). A more stereotyped adult-like pattern, including a rapidly occurring peak shortly after awakening, develops slightly later, sometime in the 12-24-month-old age range (Saridjan et al., 2010). Layered on top of this, diurnal patterns may include short-term elevations in cortisol resulting from acute stressors in the environment which can include both physical (e.g., injury) or psychosocial (e.g., unpredictable, uncontrollable, threatening) events. During infancy, separations from the primary caregivers constitute acute psychosocial stress given dependence on caregivers for food, warmth, protection, and physiological regulation (Gunnar and Donzella, 2002).

The majority of research on pre- and perinatal stress, fetal HPA activity, and substance use has been completed in animal studies; however, this is complemented by growing evidence in human research. Animals studies have outlined that exposure to pre- and perinatal stress, via fetal programming, affects adult behavior in three primary domains: learning impairment, increased anxiety and depressive behaviors, and enhanced sensitivity to drugs of abuse (Lupien et al., 2009). Animal research measuring fetal biology across many species (e.g., rodent, guinea pig, primate) have shown that single or repeated exposure to maternal stress leads to elevated maternal GC secretion, which increases fetal HPA activity via the placenta, measured by the amount and/or duration of corticosterone secretion in the offspring (Henry et al., 1994; Kapoor and Matthews, 2005; Seckl, 2007).

## 2 The HPA axis and fetal programming

Pregnancy is a particularly sensitive period of HPA axis development, in which fetal biology is influenced by the intrauterine environment, maternal HPA axis function, and fetal HPA axis development (Davis and Sandman, 2010). The “fetal origins of adult disease” hypothesis postulates that the development of some or many common adult diseases, including physical (e.g., diabetes mellitus) and mental health illnesses (e.g., SUDs), originates during the fetal period (Hellemans et al., 2010). In contrast to the negative HPA-axis feedback loop in the brain and pituitary gland, there is a positive feedback loop during pregnancy whereby cortisol stimulates placental CRH production, leading to concurrent increases of CRH, ACTH, and cortisol throughout gestation (King et al., 2001; Wadhwa, 2005). During normative gestation, maternal cortisol levels rise two-to-four fold (Brown et al., 1996); in larger quantities, cortisol is harmful to the developing fetus. Though a placental barrier enzyme provides some buffering of the fetus from maternal stress hormones, this barrier is not impermeable, with increasing fetal exposure to GCs observed during periods of chronic or severe maternal stress (O’Donnell et al., 2012). Due to the underdeveloped fetal blood-brain barrier, the fetal brain is highly susceptible to such GC exposure (Wadhwa, 2005). We explore three candidate pathways by which pre-and perinatal stress and HPA functioning might contribute to substance abuse risk: prenatal exposure to maternal stress and GCs, prenatal exposure to maternal substance use, and postnatal stress exposure.

## 3 Prenatal stress exposure, substance abuse risk, and the HPA-Axis

### 3.1 Prenatal exposure to maternal stress

Animal Studies. Animal models during the pre- and postnatal periods have investigated varying types of stress (e.g., synthetic GC administration, food restriction, restraint paradigms, maternal separation, and drug exposure). Across several stress paradigms, offspring of stress-exposed rodent dams exhibit increased sensitivity (i.e., enhanced novelty towards drugs and susceptibility to addiction) to a wide range of drugs of abuse including amphetamines (Deminière et al., 1992; Diaz et al., 1995; Henry et al., 1995), 3, 4-methylenedioxy methamphetamine (MDMA) (Morley-Fletcher et al., 2004), ethanol (Biggio et al., 2018; Rodrigues et al., 2012), morphine/opioids (Deroche et al., 1992, 1995; Rodrigues et al., 2012), and cocaine (Kippin et al., 2008; Pastor et al., 2018). However, few studies have explicitly delineated how neuroendocrine activity may mediate this pathway. Deroche and colleagues found that an adrenalectomy, which suppresses corticosterone secretion, prevented increased sensitivity to amphetamine in rodent offspring of stress-exposed mothers (Deroche et al., 1992). Additional research is required to precisely determine how neuroendocrine functioning following prenatal stress influences substance use and addiction.

Clinical Studies. In humans, research focused specifically on the intersection of prenatal stress, the HPA axis, and offspring's substance abuse is limited. The most common type of stress studied is heightened stress during pregnancy, maternal prenatal depression, and maternal prenatal anxiety. Altered HPA functioning and GR gene expression have been observed in prenatally-stressed offspring (Sosnowski et al., 2018). For example, maternal anxiety and depression in the third trimester was associated with increased methylation of a GR gene (*NR3C1*) and increased cortisol responses to stress for offspring at 3 months of age, even after controlling for postnatal maternal mood (Oberlander et al., 2008). Higher prenatal maternal depressive symptoms and lower social support also predicted higher basal cortisol levels among infants (Luecken et al., 2015). Prenatal stress exposure may also impact offspring's neuroendocrine functioning beyond infancy. A study of school-aged children exposed prenatally to maternal depressive symptoms exhibited altered methylation of the *NR3C1* gene and lower cortisol release for boys and higher cortisol release in girls (Stonawski et al., 2018). Prenatal intimate partner violence exposure was also linked to increased cortisol reactivity to stress for 11 month-13 month old infants (Levendosky et al., 2016). It should be noted that many of these stressors are co-occurring conditions. Future research should carefully consider the role of multiple types of pre- and perinatal stressors on substance abuse vulnerability in offspring.

Summary. Overall, animal studies have consistently found that prenatal stress exposure increases sensitivity towards drugs and heightens susceptibility to addiction in offspring. Preliminary evidence suggests that neuroendocrine systems may play a role in this pathway; however, additional studies are needed to further elucidate these complex interactions. In human studies, research is more limited, with no studies specifically exploring links between prenatal stress exposure and HPA-axis activity with future substance abuse risk. However, studies have found links between prenatal stress and HPA-axis activity in prenatally-stressed offspring, such as increased methylation of GR genes and altered cortisol levels and reactivity.

## 4 Prenatal exposure to glucocorticoids

Antenatal corticosteroids, a common therapy for pregnant women expecting preterm delivery, may also alter HPA-axis function in the short-term (e.g., suppression of cortisol response to stress), providing a naturalistic window to understanding stress hormones impacts on fetal development and substance abuse risk (De Blasio et al., 2007; Waffarn and Davis, 2012). In a prospective design, Van Lieshout and colleagues found that low birth weight survivors exposed to antenatal corticosteroids had the highest risk for psychiatric and substance use disorders and risk odds increased correspondingly with steroid exposure (Van Lieshout, Boyle, Saigal, Morrison and Schmidt, 2015).

### 4.1 Prenatal substance exposure

Prenatal substance exposure constitutes an extreme form of prenatal stress. In the United States, it has been estimated that approximately 2.8–4.3% of pregnant women utilize illicit drugs (Ebrahim and Gfroerer, 2003; Lester et al., 2004; Office of Applied Studies., 2005), while upwards of 12% of pregnant women use cigarettes (Lester et al., 2004). These rates are significantly higher in women living in poverty and those receiving limited prenatal care (Birnbach et al., 2001). Several issues here must be addressed. First, polysubstance abuse is more common than single-substance abuse both during and after pregnancy (Birnbach et al., 2001; Ebrahim and Gfroerer, 2003). An abundant number of animal studies have documented effects of individual drugs on fetal development, however, fewer animal paradigms have investigated impacts of multiple prenatal drug administration. In human studies, researchers will often statistically control for other drug use or recruit single-substance users, which may not be generalizable to the population. A growing number of studies have begun to specifically investigate impacts of prenatal polysubstance use on offspring development (e.g., Nygaard et al., 2016), yet, few studies have examined prenatal polysubstance abuse as a risk factor for offspring substance misuse problems.

Further, different substances have varying effects on neurobiological systems. All drugs increase levels of dopamine in the brain and there is significant overlap of the neural systems impacted by prenatal substance exposure (e.g., dopaminergic and serotonergic neurotransmitters, vasoconstriction) (as reviewed in, Ross et al., 2015). Yet, the individual mechanisms of action for each drug are important to understanding the pathways for developmental effects of polydrug use on offspring (Lester et al., 2004). In addition, the timing of prenatal drug exposure is important, as impacts on the fetus vary based on developmental stages. A comprehensive review of the differential impacts of type of substances and timing can be found elsewhere (Forray, 2016; Glantz and Chambers, 2006; Lester et al., 2004).

Animal Studies. Several animal studies have illustrated how prenatal drug exposure confers risk for future substance misuse in offspring. A review of animal paradigms of prenatal drug exposure outlines that prenatal drug exposure, similar to other stress paradigms, is linked to enhanced sensitivity and preference for drugs in offspring across several types of drug exposure (Malanga and Kosofsky, 2003). For example, cocaine was found to be more reinforcing in rats that had been prenatally exposed to cocaine. Responsivity was conceptualized as increased rates of elevated active lever responding to cocaine administration (Hecht et al., 1998; Keller et al., 1996; Kippin et al., 2008). Additionally, prenatal morphine exposure was associated with increased self-administration of cocaine and heroin in adult rats (Ramsey et al., 1993).

Clinical Studies. In humans, there is growing evidence for prenatal substance exposure predicting offspring substance use (Glantz and Chambers, 2006; Lester and Lagasse, 2010). There is substantial evidence that childhood-onset substance use is uniquely related to prenatal exposure of nicotine and alcohol (Lester et al., 2004). In this section, we will review literature on nicotine and cannabis exposure, alcohol exposure, and cocaine exposure during pregnancy.

Nicotine and Cannabis Exposure. Prenatal exposure to nicotine is associated with an increased likelihood that offspring will smoke cigarettes in adolescence (Kandel et al., 1994; Kandel and Udry, 1999), and in offspring being at higher risk for nicotine dependence (De Genna et al., 2017). Prenatal nicotine use also predicted alcohol and cannabis-use disorders in a study of over 7000 young adults (Salom et al., 2016). Maternal smoking during pregnancy was also linked to increased risk for hospitalization of offspring for substance abuse in a large longitudinal study (Brennan et al., 2002). Additionally, both cannabis and tobacco use during pregnancy have been linked to an increased risk for earlier initiation of cigarette and cannabis use in offspring (Cornelius et al., 2000; Porath and Fried, 2005).

Alcohol Exposure. With respect to alcohol, a longitudinal study found that prenatal alcohol exposure was associated with alcohol problems in offspring at 21 years of age, even after controlling for family history of alcohol problems, nicotine exposure, other prenatal stress exposures, and postnatal environmental factors (Baer et al., 2003). A separate study also demonstrated that prenatal alcohol exposure predicted increased alcohol problems, as well as psychiatric disorders, among offspring at 25 years of age (Streissguth, 2007). Notably, prenatal alcohol exposure has been a stronger predictor of adolescent drinking than a family history of alcohol problems (Baer et al., 1998).

Cocaine Exposure. Prenatal cocaine exposure has been similarly linked with adolescent use of psychoactive substances (Bennett et al., 2007; Delaney-Black et al., 2011; Frank et al., 2011; Lester et al., 2012; Min et al., 2014; Minnes et al., 2017; Yip et al., 2016), with one study finding that prenatally cocaine-exposed adolescents were 2.8 times more likely to have substance-use related problems compared to their non-exposed counterparts (Min et al., 2014).

## 5 HPA-axis and prenatal substance exposure in clinical studies

Basal Cortisol. HPA-axis alterations have promising evidence as a potential causal link, particularly as prenatal substance exposure has also been shown to impact neuroendocrine functioning. Overall, prenatal substance exposure has generally been linked to higher basal cortisol levels in infants, with some variation depending on the substance. For example, infants prenatally exposed to alcohol and cigarette use had higher basal cortisol levels compared to control infants (Ramsay et al., 1996). Cortisol levels were significantly elevated in the afternoon and bedtime for children aged 6–14 with fetal alcohol spectrum disorders who had high levels of prenatal alcohol exposure compared to control children or children with lower levels of prenatal alcohol exposure (Keiver et al., 2015). Jacobson and colleagues found that heavy drinking during pregnancy was associated with higher basal cortisol levels in 13-month-old infants; however, cocaine use was related to lower basal levels (Jacobson et al., 1999).

While most studies did not examine substance use patterns in offspring, chronic stress and the accompanying altered HPA-axis, has been proposed as a neurobiological mechanism underpinning the onset and maintenance of SUDs and addiction (Sinha, 2008). In favor of this theory, HPA-axis function has been shown to predict substance abuse vulnerability amongst prenatally drug-exposed populations. This includes evidence that highlighted discordance between parasympathetic nervous system function (supporting physiological regulation) at age 3 and baseline cortisol levels at age 11 as a predictor of earlier initiation of alcohol use in prenatally drug-exposed adolescents. In females, a discordance between RSA and cortisol (i.e., high RSA and low cortisol or low RSA and high cortisol) was linked to more executive dysfunction, which then predicted earlier initiation of alcohol use. In comparison, only the discordance of low RSA and high cortisol in boys showed the same pattern of elevated executive dysfunction leading to earlier alcohol initiation (Conradt et al., 2014). These results highlight the importance of not only investigating interactions between different aspects of physiological systems (e.g., HPA and the autonomic nervous system), but also examining related behavioral domains (e.g., executive function) to best predict substance misuse in vulnerable populations.

Cortisol Reactivity. Prenatal substance exposure has also been studied in relation to cortisol reactivity, with directionality of results linked both to age of assessment, stressor paradigm, and type of substance exposure. A study of maltreated foster care children aged 9–12 found that children exposed generally to prenatal substances were more likely to have attenuated HPA reactivity to a social laboratory stressor compared to maltreated foster care children without prenatal substance exposure (Fisher et al., 2012). Comparable findings have also been observed in prenatally drug exposed adolescents who exhibited suppressed task-related cortisol reactivity compared to a control group. Notably, cortisol reactivity mediated the association between prenatal drug exposure and drug experimentation, in that adolescents with an attenuated cortisol response had increased likelihood of drug experimentation (Buckingham-Howes et al., 2016).

On the other hand, heavy prenatal exposure to alcohol has been linked to elevated post-stress (i.e., blood draw) cortisol levels in 13-month-old infants (Jacobson et al., 1999). Prenatal alcohol exposure was also linked to greater cortisol reactivity in 5–7 month-olds following a “still face” procedure, a paradigm used to investigate stress regulation (Haley et al., 2006). Similar to prenatal alcohol exposure, cigarette exposure during pregnancy has also been linked to higher peak cortisol reactivity in 7-month-old infants following an arm-restraint paradigm (Schuetze et al., 2008) and one-month old infants following a neurobehavioral exam (Stroud et al., 2014). Of note, child sex may moderate this relationship. A study by Eiden and colleagues found an association between prenatal tobacco exposure and *blunted* cortisol reactivity, but only in boy infants (Eiden et al., 2015). Additional research is needed to further elucidate the complex relationship between prenatal tobacco and alcohol exposure and cortisol reactivity in infants.

Studies examining prenatal cocaine exposure have yielded mixed results. In a study comparing prenatally cocaine-exposed to non-exposed infants, cocaine-exposed infants had lower levels of cortisol across two types of stressor conditions, a “noninvasive” neurobehavioral examination and an “invasive” heel-stick procedure (Magnano et al., 1992). Notably, the opposite effect has also been observed in infants, with one study finding that prenatally cocaine-exposed infants exhibited greater cortisol reactivity following an emotional arousal stress paradigm (Eiden et al., 2009). Prenatally cocaine-exposed children (age 11) were less likely than non-exposed youth to show an elevated cortisol response to a stressor. Further, among prenatally cocaine-exposed children, those who experienced domestic violence were the most likely to exhibit a blunted HPA axis response, suggesting that early adversity and prenatal substance exposure might combine to strongly influence neuroendocrine functioning (Lester et al., 2010). A separate longitudinal study in low-income 14–17 year olds found that prenatally cocaine-exposed youth exhibited a blunted cortisol response to a laboratory social stressor compared to a control adolescent group without prenatal cocaine exposure (Chaplin et al., 2015). Interestingly, although cortisol reactivity to the stressor was not predictive of subsequent substance use, other markers of biobehavioral regulation in response to the psychosocial stressor (i.e., sadness, in girls; blunted sympathetic nervous system reactivity, in boys) predicted substance use at 6–12 month follow up (Chaplin et al., 2015).

Mixed results may be due to the age of the offspring, the type of stress paradigm, and differing drug exposure. Further, one study did find that hyporeactivity of the HPA-axis to a stressor was linked to drug experimentation in prenatally drug exposed youth (Buckingham-Howes et al., 2016). It has been theorized that chronic stress initially leads to HPA-axis over-activity, but that, over time, the neuroendocrine system becomes down-regulated (Miller et al., 2007). This suppression in the face of chronic stress may be a mechanism to protect bodily functions from excessive exposure to cortisol but may also have far reaching metabolic and regulatory consequences with notable links to substance use and psychopathology (Koob and Le Moal, 2001; Miller et al., 2007). Further, the HPA-axis undergoes substantial pre- and post-natal development and programming in the first 5 years of life (Glover, O'Connor, & O'Connell, 2010). Accordingly, mixed results may be indicative of the varying timepoints of stressor occurrence and neuroendocrine adaptation (Roos et al., in press). Overall, findings have begun to explore how neuroendocrine functioning may enhance substance abuse vulnerability following pre- and perinatal stress, but additional research is warranted to further elucidate these pathways.

Summary. Cumulatively, animal and clinical studies have both provided substantial evidence connecting prenatal drug exposure, a potent form of stress, to alterations in HPA-axis functioning and future substance abuse problems. In animals, prenatal drug exposure has been consistently linked to enhanced sensitivity and preference for drugs in offspring. In humans, several studies have found that prenatal alcohol and drug exposure predicts offspring alcohol and drug use. Further, clinical studies have established that prenatal drug exposure predicts higher basal cortisol levels.

Although no study has explicitly explored the pathway from prenatal drug exposure to elevated basal cortisol levels to substance abuse, Conradt and colleagues did find that a discordance between parasympathetic nervous system functioning and baseline cortisol levels predicted earlier initiation of alcohol use in prenatally drug-exposed adolescents (Conradt et al., 2014). Additionally, several studies have examined how prenatal drug exposure predicts cortisol reactivity in offspring, with mixed results highlighting both blunted and hyperactive cortisol reactivity to stressors. Future research is needed to consolidate these findings and extend them to explore pathways from prenatal drug exposure to substance abuse risk and the role of the HPA-axis.

Importantly, chronic stress may also augment drug abuse vulnerability through the co-activation of multiple stress response systems. For example, it has been proposed that a co-activation of the HPA-axis and dopaminergic pathways enhance the reinforcing properties of substances through reward circuitry alterations (Piazza and Le Moal, 1998). Documented correlations between neuroendocrine markers (e.g., cortisol) with release of dopamine in mesolimbic pathways are evidence of cross-activation between the HPA-axis and dopaminergic functioning (Sinha, 2008). Future longitudinal research is necessary to explore how neuroendocrine changes, in conjunction with related stress systems, underlie pathways to substance misuse and addiction. The complexity, and potentially sex-specific links between prenatal and perinatal experiences, across stress-system, neural, and behavioral processes, highlights the need for further longitudinal research across measurement techniques to gain a better understanding of the developmental trajectories of children at risk for later life addiction (Roos et al., in press).

### 5.1 Postnatal stress exposure, HPA-Axis, and substance abuse vulnerability

Animal Studies. In animals, maternal separation paradigms are the most utilized procedure to examine postnatal stress, namely the effects of neglect during the postpartum period on offspring development. Because newborns are fully dependent on their mothers for care and survival in many species, the prolonged or repetitive lack of maternal presence induces both behavioral and biological markers of distress across rodent, primate, and human samples (Lupien et al., 2009). Rodent research indicates that maternal separation is associated with a predisposition for higher ethanol consumption and/or preference in adult rats, particularly in male offspring (Nylander and Roman, 2013).

A rodent study on postnatal maternal separation found that offspring demonstrated increased vulnerability to ethanol consumption during adolescence with enhanced expression of key genes associated with HPA axis functioning (de Almeida Magalhães, Correia, de Carvalho, Damasceno and Brunialti Godard, 2017). A separate rodent study found that maternally separated offspring demonstrated a strong preference for ethanol, elevations in ACTH and corticosterone during a lab stressor, and that the ethanol consumption was correlated with the stress response (Huot et al., 2001). Notably, paradigms that combine effects of prenatal exposure to stress and drugs have shown the dual impacts of substance use and stress on development. For example, a rodent study demonstrated that gestational ethanol exposure, combined with maternal separation during the postpartum period, predicted heightened anxiety, while postpartum exposure to maternal separation alone predicted an enhanced preference for ethanol (Biggio et al., 2018). Of note, rodents exposed only to prenatal ethanol exposure did not exhibit this enhanced preference for ethanol (Biggio et al., 2018). Overall, maternal separation is undoubtedly a risk factor for substance use in animal models, providing theoretical frameworks to help understand the impacts of postnatal stress on the development of alcohol and drug preference and use in offspring (as reviewed in, Moffett et al., 2007).

Clinical Studies. Clinical studies in humans have begun to build off of well-known findings from the animal literature. Research has shown that mothers experiencing prenatal stress are at heightened risk for abusing substances during and after pregnancy (Barnet et al., 1995; Kingston et al., 2016; Zambrana et al., 1997). Comparable to prenatal stress exposure, studies have yielded mixed findings on the relationship of postnatal stress to infant cortisol reactivity. For example, in one study, infants of postnatally depressed mothers exhibited more negative emotionality, less mature regulatory behaviors in response to fear, and higher cortisol reactivity compared to a control group (Feldman et al., 2009). Other studies have found infant cortisol hyperactivity to a stressor. One study found that maternal child abuse was associated with infant HPA axis function (i.e., lower baseline cortisol levels during a stress paradigm) during the postpartum period (Brand et al., 2010). More research is needed to delineate how postnatal stress impacts neuroendocrine functioning in infants as well as exploring associations between postnatal stress, neuroendocrine functioning, and addiction vulnerability.

Few studies have explored links between postnatal drug and alcohol use and offspring's substance abuse risk. However, this is an important area of research as studies have consistently shown that while many women do successfully moderate alcohol and drug use during pregnancy, they often relapse or increase substance use in the postnatal period (Forray and Foster, 2015). Postnatal drug use is a potent form of early life stress, as offspring of women with postnatal substance use problems are frequently exposed to several other stressors (e.g., poor nutrition). Additionally, mothers are at higher risk for disrupted parental care and dysfunctional attachment with offspring (as reviewed in, Forray and Foster, 2015). One study did find that prenatal and postnatal cocaine use were both uniquely related to adolescent offspring use of cocaine (Delaney-Black et al., 2011).

Summary. Most animal studies have utilized a maternal stress paradigm to investigate postnatal stress and impacts on neuroendocrine functioning and offspring risk for substance misuse and addiction. These studies have found that offspring exhibit a stronger preference and higher rates of consumption of ethanol. They have also established preliminary links with altered HPA-axis functioning, such as elevations in ACTH and corticosterone, which are linked to ethanol consumption, for maternally-separated offspring. Further, animal studies have begun to document interactive effects of prenatal substance exposure and postnatal stress (e.g., Biggio et al., 2018). As most children are exposed to more than one type of stressor, such studies are important, and more research is required to delineate the potentiating impacts of prenatal and postnatal stress/substance use exposure. In human studies, postnatal stress, such as maternal depression, has been linked to both hypo- and hyper-cortisol reactivity. More research is required to establish if such alterations in the infant predict future substance abuse problems.

### 5.2 Early experiences, the immune system, and substance abuse

Until recently, pregnancy was conceptualized as a continuously immune-suppressed state (Mor et al., 2017; Wadhwa et al., 2001). However, recent evidence suggests that the immune system during pregnancy is multifaceted and dynamic, suppressed at times but elevated at others, in which a critical interplay exists between fetal cells and the mother's immune response (Jennewein et al., 2017). During implantation of the embryo, trophoblast cells from the embryo invade and attach to the womb's lining, leading to the formation of the placenta, and triggering an inflammatory cascade (Mor et al., 2017). The inflammatory response is necessary for successful implantation and a pro-inflammatory state dominates the first 12 weeks of pregnancy (Mor et al., 2017). During the following 15 weeks, an ensuing period of rapid fetal growth and development when hyper-inflammation can contribute to miscarriage, preterm birth, and other deleterious outcomes, the mother, placenta, and fetus are immunosuppressed (Mor et al., 2017; Romero et al., 2007). At the end of pregnancy, there is a shift to a pro-inflammatory state during labor and delivery (Mor et al., 2017). Importantly, during the first and second trimester, maternal antibodies are transferred across the placenta, and after birth through breast milk. Maternal antibodies help infants form passive immunity to pathogens and modulate the infant's immune responses (Jennewein et al., 2017).

## 6 HPA axis and the immune system

The HPA axis and immune system interact in important ways during pregnancy. Glucocorticoids regulate several properties of immune cells, including activation, differentiation, growth and apoptosis (Dhabhar and McEwen, 2001; Oppong and Cato, 2015). A growing area of study is how prenatal stress can disrupt the placental transfer of maternal antibodies to confer risk for dysregulated immune functioning in offspring and what role GCs might play in that disruption. Elevated maternal GCs can alter placental functioning, an important site of immune symbiosis between mother and fetus (Merlot et al., 2008). In primates, chronic social stress has been found to significantly alter antibody levels in mothers and neonates, with male offspring born with lower IgG levels and female infants having higher-than-usual IgG levels (Coe and Crispen, 2000). It has been theorized that GCs are mediators for the impacts of prenatal stress on immune functioning, though research on this is mixed (Merlot et al., 2008). It is likely the case that GCs, and the HPA axis, are among several mediators between prenatal stress and immune functioning (Merlot et al., 2008).

### 6.1 Prenatal and perinatal stress, immune functioning, and substance use

#### 6.1.1 Animal studies

Prenatal Stress. In animal studies, a wide range of prenatal stressors, including prenatal immune activation, viral infection, psychological stress, and malnutrition, have been linked to the development of behavioral abnormalities in offspring that often co-occur with addiction, such as deficits in information processing, sensorimotor skills, cognition, social functioning, and the onset of depressive phenotypes (Markham and Koenig, 2011).

Using rodent models, early studies found that prenatal stress suppressed immune functioning as measured by several indices, such as rate of proliferation of lymphocytes, the body's response to infections (e.g., herpes simplex virus-type 1 injection), and circulating pro-inflammatory cytokine levels (Merlot et al., 2008). Overall, administration of GC treatment during pregnancy generally inhibits immune functioning while stress paradigms have yielded more inconsistent results, with some indicating that stress enhances immune activity while others finding the opposite effect (as reviewed in, Merlot et al., 2008). Discrepancies could be due to differing paradigms (e.g., types of stress), animal type (e.g., rodent, pig, monkey), and timing of the stressor during gestation. Additionally, in animal studies (e.g., rodent, mouse), prenatal immune activation has been linked to increased dopamine release (Vuillermot et al., 2010; Zuckerman et al., 2003). A longitudinal study with mice found evidence for maladaptive dopaminergic functioning (e.g., an increase in dopamine cells) in offspring as early as the fetal stages following a prenatal immune challenge that simulates a viral infection. Such findings have strong implications for the role of the immune system in addiction vulnerability as dopaminergic systems are also key to the neurobiology of substance abuse (Grant et al., 2006).

#### 6.1.2 Clinical Studies

In humans, immune alterations during pregnancy adversely impact the offspring and increase risk for immune-related ailments in children. For example, maternal exposure to infection during pregnancy is associated with risk for several immune-related physical ailments in offspring, such as asthma and allergies (Flanigan et al., 2018). The extant literature on prenatal stress and immune functioning is limited in human samples. One study found that anxiety during pregnancy was linked to poorer adaptive immune response in infants at 6 months, as measured by both dampened responses to immunizations (i.e., reduced hepatitis B antibody titers following immunization) and dysregulated responses to antigens (i.e., increased interleukin-4 and decreased interferon gamma responder cell frequencies in response to an antigen) (O'Connor et al., 2013). Maternal prenatal anxiety has also been associated with an altered immune response to antigens in other studies (Ota et al., 2004; PrabhuDas et al., 2011). Recently, a study linked maternal prenatal depression to greater infant negative affect and this association was mediated by increased maternal cytokines during the third trimester (Gustafsson et al., 2018). Overall, emerging evidence supports the hypothesis that prenatal stress impacts infant's immune functioning with important implications for risk factors related to addiction risk.

#### 6.1.3 Prenatal drug exposure

Animal Studies. A small body of studies has explored prenatal drug exposure and immune functioning. For example, pregnant rats that were injected with cocaine experienced an increase of cytopathic damage following a herpes simplex virus-type 1 injection, indicative of an underperforming immune system (Sobrian et al., 1990). Prenatal immune activation (e.g., exposure to infection) in rodent models has also been linked to enhanced sensitivity to the locomotor stimulating effects of amphetamine in offspring (Zuckerman et al., 2003) and increased sensitivity to the stimulant effects of amphetamine in offspring (Borçoi et al., 2015; Meyer, Engler, Weber, Schedlowski and Feldon, 2008a; Meyer, Nyffeler, Yee, Knuesel and Feldon, 2008b). It is important to note that these findings derive from a model of schizophrenia; nevertheless, they provide initial evidence that prenatal immune activation alters amphetamine-related behaviors and therefore can be applied to the context of substance addiction vulnerability. Further, substance abuse is commonly comorbid in schizophrenia (Schuckit, 2006; Swofford et al., 1996).

Clinical Studies. Newborns whose mothers used crack or cocaine during pregnancy have elevated inflammation in umbilical cord blood compared to control children, even after adjusting for maternal psychopathology, sociodemographic factors, and nicotine consumption (Mardini et al., 2016). Maternal alcohol consumption during pregnancy increases the risk for infection during early childhood due to alcohol-induced reductions in immune responses to antigens (Pasala et al., 2015). Maternal smoking during pregnancy has also been linked to increased levels of cytokines, such as interleukin-8, in newborns. Of note, this relationship was not observed in the neonates of mothers who ceased smoking at any point during pregnancy (Chalal et al., 2017).

In terms of stress-immune interactions, initial evidence suggests that prenatal and perinatal stress may be linked to elevated inflammation even later in life. Adult women whose mothers reported psychosocial stress during pregnancy had elevated pro-inflammatory cytokine levels (Entringer et al., 2008). Prenatal and postnatal stressors have also been linked to elevated inflammation in adult offspring (Pedersen et al., 2018). Though a potential causal role of immune dysregulations in psychological disorders, such as major depressive disorder, has garnered significant recent attention, less research has focused on immune effects on substance use and addiction. The high rate of comorbidity between SUDs and psychological disorders, notably depressive disorders, warrants additional research on how immune dysregulations might increase vulnerability for addiction (Swendsen and Merikangas, 2000). Further, initial evidence does suggest a role for immune dysregulation in drug addiction (Fleischer et al., 2017).

HIV Exposure. Exposure to HIV during pregnancy also provides a naturalistic window to exploring how perinatal immune disruptions may impact offspring. Individuals with HIV have a weakened immune system, as the virus destroys white blood cells that fight infection. Substance use is often comorbid with HIV, an issue that is perpetuated by prenatal exposure to HIV (Sullivan et al., 2015). A study of 530 HIV-infected pregnancy women in the US found that 42% used “hard drugs” during pregnancy (i.e., cocaine, opioids, heroin/methadone) (Rodriguez et al., 1996). Further, hard drug use was associated with greater likelihood of HIV transmission to the offspring (Rodriguez et al., 1996). Perinatal HIV exposure has been linked to poorer mental health (Mellins et al., 2009, 2011) (Mellins et al., 2009, 2011) and higher rates of risky sexual behavior in adolescents (Elkington et al., 2009; Mellins et al., 2011). In turn, in these studies, adolescent substance use was then linked to poorer mental health (Mellins et al., 2009) and sexual risk behaviors (Elkington et al., 2009), suggesting that perinatal HIV exposure promotes risk to maladaptive outcomes (e.g., mental health, sexual risk behavior) that may, in turn, compound risk for substance use.

Studies on perinatally HIV-infected (PHIV) youth have established links between perinatal exposure with substance abuse. For example, substance use symptoms increased over time only for PHIV infected youth compared to PHIV-exposed but non-infected youth in a longitudinal study (Mutumba et al., 2017). In a separate study, PHIV youth had smaller grey matter volume compared to HIV-unexposed youth and alcohol and marijuana use were associated with even greater reductions of brain volume (Lewis-de los Angeles et al., 2017). Notably, lower grey matter volume has been documented in individuals with SUDs and more severe grey matter deficits are reported in relapsers (Sinha, 2011). Altogether, compelling evidence suggests that perinatal HIV exposure confers risk for substance abuse and related outcomes; however, more research is needed to elucidate how the immune system following HIV-exposure specifically mediates or moderates these pathways.

Summary. Altogether, emerging research has implicated how prenatal and perinatal stress harms immune function. Taken together with studies that have implicated immune dysregulations in the neurobiology of SUDs and addiction and related constructs, an important future direction for the field will be to examine if pre- and perinatal stress-induced immune alterations serve as a neurobiological pathway from early life stress to substance abuse and addiction. Initial evidence in animal models have shown that a form of prenatal stress, prenatal immune activation, increased sensitivity to the simulating effects of certain drugs, such as amphetamine. In clinical studies, links between prenatal and perinatal stress and inflammation have been established. Further, prenatal drug and HIV exposure have both been linked to substance misuse problems in offspring.

### 6.2 Future directions

A complex combination of genetic, environmental, neurobiological, and psychological factors contribute to vulnerability to drug addiction (Kreek et al., 2005). Prenatal and perinatal stress are potent environmental factors that disrupts stress systems and enhances offspring risk of substance abuse and addiction.

Substantial progress has been made in animal and clinical research to delineate the impacts of prenatal and perinatal stress on offspring's neurobiological systems and drug abuse vulnerability, but there is a critical need for longitudinal research to investigate mechanistic links between early neurobiology with later addiction. An emerging challenge for the field is to find new ways to translate knowledge about the pathways linking the earliest forms of stress to risk for substance abuse and addiction. A translational neuroscience framework places emphasis on designing and tailoring interventions in a way that is informed by a nuanced understanding of the multifarious effects of pre- and perinatal stress and its downstream impact on addiction vulnerability.

Here, we highlight important areas for future study and significant limitations. As noted previously, it is more common that multiple stressor types co-occur, such as elevated prenatal maternal psychopathology in the context of IPV (Howard et al., 2013) or prenatal polysubstance abuse (Ebrahim and Gfroerer, 2003). Additionally, there may be potentiating effects of prenatal stress when combined with postnatal stressors (e.g., Biggio et al., 2018). However, these phenomena are often studied separately, limiting the understanding of the combined effects of pre- and postnatal stress. Health disparities, such as SES and minority status, are another significant consideration. Poverty, minority status, and mental health pathology often co-occur, constituting a severe stressor for the offspring (Nagahawatte& Goldenberg, 2008). PHIV + youth are a particularly vulnerable population, as they are more likely to be exposed to poverty, racism and discrimination, parental substance use, and neighborhood violence compared to non-exposed youth (Mellins et al., 2011). More research is needed to carefully examine concomitant stressors on offspring development and addiction vulnerability. Such work will help to both understand individual impacts on certain types of stressors, as well as the additive effects of polyvictimization on offspring for addiction vulnerability.

In conjunction with neurobiological systems, there are several other important moderators to consider in the pathway linking pre- and perinatal stress to offspring addiction vulnerability. Quality of parental care, such attachment and warmth, may contribute to individual differences in offspring exposed to fetal stress. Pre- and perinatal stressors are linked to lower levels of maternal warmth and dysregulated attachment with the infant (Quinlivan and Evans, 2005), which in separate studies, are linked to substance abuse and addiction (Schindler et al., 2005). Dysregulated attachment styles are also associated with elevated cortisol (Ahnert et al., 2004) and inflammation in children (Measelle et al., 2017). Further, later stages of child development are also significant as they contribute to parenting capacities and may include additional windows of developmental plasticity. Pre- and perinatal stress may lead to poorer stress functioning in young offspring (Gutteling et al., 2005), as neurobiological disruptions to neuroendocrine and immune pathways become biologically embedded. In turn, such behavioral dysfunction may further interact with parenting capacities to increase the deleterious impact of such pre- and perinatal stressors.

Interactive associations between parenting and child stress response have also been observed with implications for key developmental pathways to substance abuse, such as child externalizing or conduct problems. For example, studies have found that skin conductance level reactivity, a physiological biomarker for children's stress response, moderates the association between harsh parenting and child externalizing problems (Erath et al., 2009, 2011). Cortisol reactivity may also moderate associations between quality of parenting and conduct problems. One study found that maternal sensitivity at six months predicted fewer conduct problems when the children were in the first grade, but only for children who had demonstrated high levels of cortisol reactivity following fear and frustration tasks in infancy (Wagner et al., 2017). Poor parental monitoring has also been linked to flatter morning-to-evening cortisol slopes, which in turn, were related to higher levels of externalizing behaviors in preadolescent children (Martin et al., 2014).

Altogether, there is growing evidence to support that quality of parenting is linked to child behavior problems that are known to contribute to increased risk for substance use. A growing area of research is dedicated to exploring interactions between child stress response and quality of parenting in the context of pre, peri- and postnatal to developmental trajectories that are related to addiction risk. Notably, one study did find that infants exposed to prenatal tobacco use demonstrated hypo-reactive cortisol responses to a frustration task, and that this was moderated by maternal intrusiveness, such that infants whose mothers had higher levels of intrusiveness, had a more significantly blunted cortisol reactivity (Eiden et al., 2015). A recent study exploring prenatal mood disturbances and executive functioning in school-age children found that cortisol reactivity mediated the association between depressed and/or anxious prenatal maternal mood and executive functioning, but only in boys. Specifically, prenatal maternal mood was negatively associated with children's executive functioning via heightened cortisol reactivity to a lab stress challenge (Neuenschwander et al., 2018).

Further, later stages of development, particularly adolescence, contribute to stress response system development and may also be a significant factor in the pathway from pre- and perinatal stress to later substance abuse vulnerability (Lupien et al., 2009). In fact, associations have been reported between observed parenting behavior and adolescent inflammation, such that higher frequency of positive parental behaviors was linked to lower levels of systemic inflammation (Byrne et al., 2017b) while higher levels of poor parental monitoring have been linked to increased inflammation (Byrne et al., 2017a). Future research will help elucidate how these pathways confer risk for addiction and explore the importance of quality of parenting at various stages of development, particularly for children who have been exposed to pre, peri-, and postnatal stress.

Given the importance of parenting as a moderator, parenting interventions represent an important prevention strategy for addiction vulnerability. A review of six attachment-based parenting interventions for drug-dependent mothers and young children (between birth-5 years of age) found that results were mixed on improving parent-child relationship attachment patterns, but promising in terms of enhancing maternal adjustment (e.g., reducing parenting stress) (Suchman et al., 2006). However, these studies have not documented improvements in substance use outcomes in the mothers, which may continually undermine mothers’ stress responses and parenting, and likely continue to significantly impact offspring (Suchman et al., 2006). Other parent training therapies have also been found to influence stress responsivity systems in the offspring (as reviewed in, Roos et al., in press). In light of the associations between the stress response system and elevated risk for substance use, such programs are promising candidates to become preventive interventions for substance use. A combination of developing more precise strategies for interventions and enhancing knowledge about who responds to what will ultimately yield more successful strategies that improve child trajectories and curtail the development of drug abuse and addiction.

## 7 Conclusion

The pre- and perinatal period is among the most sensitive and critical periods of development, making stress during this window a particularly robust form of adversity. Across animal and human studies, we reviewed compelling evidence for links between pre- and perinatal stress and dysregulation of the HPA-axis and immune systems. We have also presented studies highlighting the links between stress response systems and substance abuse and addiction. While less research has been done to explore pre- and perinatal stress and substance abuse risk, and their interactions with stress responsivity, emerging evidence emphasizes that this is an important area of study. Future work is needed to fully understand how the earliest changes to neuroendocrine and immunological systems predispose an individual to higher risk for substance abuse and addiction in order to strengthen theoretically-driven and empirically supported prevention efforts for substance abuse and addiction.

## Appendix A Supplementary data

Data Profile Data Profile
